# Comparison of rRNA-based reverse transcription PCR and rDNA-based PCR for the detection of streptococci in root canal infections

**DOI:** 10.1590/1678-7757-2018-0256

**Published:** 2019-07-29

**Authors:** Laís Cunha PRADO, Giulio GAVINI, Amanda da Costa SILVEIRA, Vitor Cesar NAKAMURA, Marcia Pinto Alves MAYER, Ericka Tavares PINHEIRO

**Affiliations:** 1 Universidade de São Paulo, Faculdade de Odontologia, São Paulo, SP, Brasil.; 2 Universidade de São Paulo, Faculdade de Odontologia, Instituto de Ciências Biomédicas, São Paulo, SP, Brasil.

**Keywords:** Streptococcus, Reverse transcription polymerase chain reaction, Polymerase chain reaction, Root canal treatment

## Abstract

**Objective:**

The rDNA-based method is unable to distinguish between alive and dead cells. Alternatively, bacterial viability can be assessed by molecular methods based on ribosomal RNA (rRNA). Therefore, this study aimed to detect viable streptococci in root canal samples using rRNA-based reverse transcription polymerase chain reaction (RT-PCR), compared to an rDNA-based PCR assay.

**Methodology:**

Microbiological root canal samples were obtained from 32 teeth with primary endodontic infections before (S1) and after chemomechanical preparation (S2), and after removal of intracanal medication (S3). RNA and DNA were extracted, and complementary DNA (cDNA) was synthesized from RNA using RT reaction. cDNA and genomic DNA were subjected to PCR with primers complementary to the 16S rRNA sequences of *Streptococcus* spp. McNemar’s test was used to compare the detection rate of both assays (P<0.05).

**Results:**

Streptococci were detected in 28.12% (9/32) and 37.5% (12/32) of S1 samples using rRNA- and rDNA-based PCR assays, respectively. In contrast, they were detected in only 6.25% (2/32) of S2 samples using rRNA-based RT-PCR, compared to 15.62% (5/32) using rDNA-based PCR. Finally, in S3 samples, streptococci were not detected by rRNA, whereas rDNA-based PCR still detected the bacteria in 12.5% (4/32) of the samples. The total number of PCR-positive reactions in the rDNA-based PCR was higher than in the rRNA-based assay (P<0.05).

**Conclusions:**

The rRNA-based RT-PCR showed a lower detection rate of streptococci when compared to the rDNA-based PCR, suggesting that the latter may have detected dead cells of streptococci in root canal samples.

## Introduction

Molecular assays targeting ribosomal DNA genes (rDNA) have been extensively used in endodontic microbiology studies.^1-9^ These methods are sensitive, fast, and allow the detection/quantification of endodontic infectious agents.^10^ However, one limitation of the rDNA-based method is the inability to distinguish between live and dead cells, which may result in an overestimation of bacterial targets in root canals, especially in post-treatment samples.^11,12^ Bacterial viability may thus be assessed by molecular methods based on ribosomal RNA (rRNA).

Ribosomal RNA can be considered an indicator of microbial viability because they degrade more rapidly than rDNA after cell death.^10^ Moreover, since the number of ribosomes per cell correlates with bacterial growth rate, rRNA-based methods are considered highly sensitive for detecting active bacterial cells in a community.^13-15^ Since the microbial community profile of root canals comprises many uncultivated bacterial species, rRNA-based molecular methods can be expected to grow in importance as instruments used to monitor viable bacterial loads during endodontic treatment. However, little evidence exists about bacterial activity in endodontic microbial communities using rRNA-based methods.^3,15^

Recently, we have compared the sensitivity of rRNA-based reverse transcription quantitative polymerase chain reaction (RT-qPCR) and rDNA-based qPCR assays for the detection of *Enterococcus faecalis* in persistent/secondary endodontic infections.^15^ In that study, rRNA-based RT-qPCR was more sensitive than the rDNA-based assay, showing that *E. faecalis* may persist active in treated root canals.^15^ Considering that bacterial species may show differences in their metabolic activity after endodontic treatment procedures, more data are needed on the activity of persistent bacterial species.

*Streptococcus* species are frequently detected in root canal samples taken after endodontic treatment procedures.^2-7^ Based on our previous study,^15^ we hypothesized that an rRNA-based molecular assay would give a better understanding of cellular viability detection rate of streptococci in root canal samples. Therefore, this study aimed to compare the detection rate of streptococci in root canal samples using rRNA-based RT-PCR and rDNA-based PCR.

## Methodology

### Patient selection

Thirty-two teeth were selected from patients seen at the graduate clinic for root canal treatment. The following inclusion criteria were considered: patients who had asymptomatic teeth with necrotic pulps confirmed by negative responses to pulp sensitivity tests; radiographic evidence of apical periodontitis in single rooted teeth or in one root with a single canal from multi-rooted teeth. Exclusion criteria were also applied, as follows: teeth from patients who had received antibiotics in the previous 3 months or who had any general disease, teeth that could not be properly isolated with rubber dam, non-restored teeth, teeth with periodontal pocket depth >4 mm; and radiographic evidence of previous endodontic treatment, open apex, crown/root fracture, root resorption, or calcification. This study was conducted in accordance with the Helsinki declaration and was approved by the appropriate Research Ethics Committee (#428.730). All patients signed an informed consent form prior to starting the study.

### Clinical and sampling procedures

Teeth were isolated with rubber dams, and the operative field was disinfected with 30% H_2_O_2_ (v/v) and 2.5% sodium hypochlorite (NaOCl) for 30 s each, followed by 5% sodium thiosulfate to inactivate the disinfecting agents. Access cavities were prepared with sterile high-speed diamond burs irrigated with sterile saline to remove caries and restorations. Before entering the pulp chamber, access cavities were disinfected again, and a bacteriological sample was taken with sterile paper points as a control sample to check the sterility of the disinfected surface. Following, access cavities were completed using new sterile diamond burs. Control samples were placed in cryotubes containing 300 µL of RNAlater solution (Life Technologies, Carlsbad, CA, USA) and frozen at -20°C for further DNA extraction. The absence of bacteria in the control sample was verified by PCR using universal primers for the *Bacteria* domain as previously described.^15^

Following access cavity preparation, the root canal was filled with sterile saline solution, and the working length was established 1.0 mm short of the apical foramen, using an electronic apex locator (J. Morita Brazil, São Paulo, SP, Brazil). Next, a #15 H-file was pushed against the root canal walls to suspend bacteria into the solution. Five sterile paper points were placed individually inside the root canal for 1 min each to collect the initial bacteria content (S1). Both the paper points and the H-file, without the handle, were transferred to cryotubes containing 300 µL of RNAlater solution (Life Technologies, Carlsbad, CA, USA) and frozen at -80°C. In each case, a single root canal was sampled to confine the microbial evaluation to a single ecological environment.

Root canal preparation was performed with R40 or R50 Reciproc instruments (VDW GmbH, Munich, Germany), depending on the initial diameter of the root canal. The selection of instruments to be used followed the manufacturer’s instructions. All instruments were used only once and then discarded. Each canal received an initial flush with 10 mL of 2.5% NaOCl delivered by a disposable syringe and 30G side-vented endodontic needles (EndoEZE, Ultradent Products Inc., South Jordan, UT, USA); next, the Reciproc instrument was inserted into the cervical third with an in-and-out pecking motion. If more pressure was needed to advance the instrument further into the canal after one cycle of three in-and-out movements, the file was removed, and its flutes were cleaned. The root canal was irrigated again with 10 mL of 2.5% NaOCl, and a new cycle of three in-and-out movements was performed in the middle third, followed by new irrigation. The instrument was inserted up to the working length with a brushing motion against the root canal walls. At the end of the preparation, the canal was irrigated with 10 mL of 2.5% NaOCl, for 40 mL volume in total. The canal was then dried using paper points and flushed with 5 mL of 5% sodium thiosulfate for 1 min. Finally, the root canal was filled with sterile saline, and a post-instrumentation sample (S2) was taken as described above.

Following, the root canal was irrigated with 2.5% NaOCl and 17% EDTA, dried using paper points, and filled with Calen paste (S.S. White, Rio de Janeiro, RJ, Brazil), comprised of Ca(OH)_2_, zinc oxide, colophony (pine resin), and polyethylene glycol 400. The paste was inserted in the canals using an ML endodontic syringe (S.S. White) attached to a Septojet XL needle (Septodont Brasil Ltda., Barueri, SP, Brazil). Radiographs confirmed proper filling of the root canals with the intracanal medication. Access cavities were filled with 2 mm of temporary restorative material (Dentalville, Joinville, SC, Brazil) and glass ionomer cement (Vidrion R, S.S. White, Rio de Janeiro, RJ, Brazil).

After 14 days, the tooth was isolated, the temporary restoration removed, and disinfection procedures of the operative field were performed following the same protocol used in the first visit. A new control sample of the dental crown and dentin surrounding the pulp chamber was obtained. The intracanal medication was removed with 10 mL of 17% EDTA and agitation with 15 K-files. Then, a third sample (S3) was taken following the same procedures described previously. Completion of the root canal treatment proceeded with root filling using lateral condensation of gutta-percha and AH Plus sealer (Dentsply Maillefer, Ballaigues, Switzerland). Access cavities were restored with temporary endodontic cement and composite resin (Z350, 3M Corporation, Saint Paul, MN, USA), and a final radiograph was taken.

### RNA and DNA extraction

RNA and DNA extraction was performed with the MasterPure Complete DNA and RNA purification kit (Epicentre Technologies, Madison, WI, USA) according to the manufacturer’s instructions. After centrifugation at 10,000×*g* for 10 min, supernatants were discarded, and pellets were re-suspended in a solution containing 300 µL of tissue and cell lysis solution and 2 µL of 50 µg/µL proteinase K. After incubation for 15 min at 65°C, the mixtures were cooled on ice for 5 min and added to 200 µL of MPC protein precipitation reagent. Following centrifugation at 10,000×*g* for 10 min, supernatants were collected and subjected to isopropanol precipitation. Total nucleic acid samples were re-suspended in 35 µL of TE buffer and divided in two vials. One vial comprised the total nucleic acid sample, which was used for DNA analysis. In the other vial, DNA was eliminated from total nucleic acid preparation by DNAse treatment and RNA purification was performed following the manufacturer’s protocol (Epicentre Technologies, Madison, WI, USA). An additional DNAse treatment was performed using DNase I (Invitrogen Corporation, Carlsbad, CA, USA), and the absence of contaminating DNA in RNA samples was confirmed by PCR. RNA and DNA concentrations were measured using a NanoDrop ND 1000 Spectrophotometer (Thermo Fisher Scientific, Wilmington, DE, USA). DNA was stored at -20°C until use. RT reactions were performed on the same day of extraction.

### Reverse transcription

The synthesis of complementary DNA (cDNA) was obtained by RT using the SuperScript^®^ III First-Strand Synthesis System following the manufacturer’s instructions (Invitrogen Corporation, Carlsbad, CA, USA). cDNA was synthesized using 8 μL of the RNA sample, random hexamers, and cDNA synthesis mix for 20 μL in total volume; cDNA was stored at -20°C until use.

### PCR

PCR reactions were performed in 50 μL total volume containing 1.25 U *Taq* DNA polymerase (Perkin-Elmer, Foster City, CA, USA), 5 μL 10× PCR buffer plus 3 mM MgCl_2_, 1 uM of each primer (*Forward* 5’-AGA GTT TGA TYM TGG CTC AG-3’ and *Reverse* 5’-TTA GCC GTC CCT TTC TGG T-3’) and 0.2 mM (each) deoxynucleoside triphosphates. For each sample, 2 μL of DNA or cDNA were added to the reaction mixture. DNA extracted from the *Streptococcus mutans* strain UA159 was used as positive control, and the reaction mixture without DNA was the negative control. Samples were subjected to 30 cycles of 94°C for 1 minute, 55°C for 1 minute, and 72°C for 2.5 minutes, plus a final 72°C extension for 10 minutes. Procedures were conducted in an automated thermal cycler Step One Plus (Applied Biosystems, Foster City, CA, USA).^2,16^

PCR products were analyzed by 1% agarose gel electrophoresis, stained with ethidium bromide, and viewed under ultraviolet transilluminator light. Positive or negative identification was based on the presence of clear bands of the expected molecular size (502 bp) using a 1-Kb lambda DNA ladder (Invitrogen Corporation, Carlsbad, CA, USA). All assays were repeated, and any cases of disagreement were repeated once again.

### Statistical analysis

McNemar’s test was used to compare the detection rates of rRNA- and rDNA-based PCR assays. Intragroup analysis was performed using Fisher’s exact test to compare the number of positive PCR reactions for streptococci in the different root canal samples. Differences were considered statistically significant when *P*<0.05.

## Results

All control samples were PCR-negative, which indicated no bacterial contamination. Streptococci were detected in 28.12% (9/32) and 37.5% (12/32) of S1 samples using the rRNA- and rDNA-based PCR assays, respectively. In contrast, they were detected in only 6.25% (2/32) of S2 samples using rRNA, compared with 15.62% (5/32) using rDNA. At the end of treatment (S3), streptococci were not detected by rRNA-based PCR, whereas rDNA was still detected in 12.5% (4/32) of the samples. Intragroup analysis revealed that the number of positive PCR results significantly decreased after chemomechanical preparation in both rRNA and rDNA groups (*P*=0.04 and *P*=0.02, respectively). However, no significant differences were observed when comparing the number of positive PCR results in S2 and S3 samples (both with *P*>0.05) (Table 1). Considering all root canal samples, the detection rate obtained by the rDNA-based PCR assay was markedly higher than that obtained by the rRNA-based assay (*P*=0.002).

**Table 1:** Number of positive PCR results for streptococci in samples taken before (S1) and after (S2) root canal preparation, and after calcium hydroxide medication (S3), using rRNA- and rDNA-based PCR assays

Samples	S1	S2	S3
rRNA	9/32 (28.12)^a^	2/32 (6.25)^b^	0^b^
rDNA	12/32 (37.5)^a^	5/32 (15.62)^b^	4/32 (12.5)^b^

Data presented as number of positive samples/number of samples analyzed (percentages)

Symbols (a, b) indicate that there were significant differences between S1 and S2 in both rRNA and rDNA groups (Fisher’s signed rank test, P<0.05)

## Discussion

Reducing viable bacterial load in root canals is a key element for endodontic treatment success. Because ribosomal RNA (rRNA) is an indicator of bacterial viability, we used an RT-PCR assay targeting 16S rRNA sequences for the detection of viable streptococci in root canal samples before and after endodontic procedures. Furthermore, we used the rDNA from the same samples as templates for PCR reactions to allow a direct comparison of the two methods.^14,15^ Due to the abundance of ribosomes present in live cells, the rRNA-based method would be expected to be more sensitive to detect viable cells when compared to the rDNA-based method.^13-15^ However, this study revealed higher streptococci detection frequencies with rDNA-based PCR, suggesting that DNA based methods might have detected non-viable streptococci after endodontic procedures. Our data support previous assertions that DNA from dead bacteria may be a potential confounder in endodontic microbiology studies, as they may overestimate the number of species identified in persistent endodontic infections.^17,18^

The genus *Streptococcus* was selected for this study because they are frequently found in primary and persistent endodontic infections in rDNA-based molecular studies.^3-6,8^ In this investigation, *Streptococcus* detection rates were assessed using both rRNA-based RT-PCR and rDNA-based PCR. Before treatment (S1 samples), detection frequencies were similar using both methods, except for three cases in which streptococcus rRNA could not be detected in rDNA-positive samples, indicating that these organisms are usually active in the microbial community of primary endodontic infections. The rRNA-negative and rDNA-positive samples before treatment suggest that streptococci may have participated in the early stages of infection in these cases, but were not active at the moment of root canal sampling. Our findings corroborate previous *in vitro* data that showed that DNA may persist in infected teeth after bacterial death.^11,17,18^ The high binding affinity between DNA and hydroxyapatite/dentin has been shown to make it less susceptible to degradation by serum and nucleases, contributing to long-term persistence of DNA in teeth.^11^

Following chemomechanical preparation (S2 samples), *Streptococcus* rDNA was detected in 5/32 canals (15.62%), which is comparable to the prevalence reported by previous rDNA-based molecular studies.^2,4,5^ However, most samples in this study that tested positive in the rDNA-based PCR assay resulted negative in the rRNA-based analysis. Our findings confirm that the detection of DNA from recently dead microbial cells may hinder a reliable analysis of antimicrobial treatment effects when using DNA-based molecular methods.^17^

The PCR detection rate in this study was higher than that of RT-PCR. This finding is in contrast with our previous study, in which the rRNA-based molecular assay was more sensitive to detect *E. faecalis* in root canals.^15^ The differences between the two studies may be related to the molecular methods used for nucleic acid detection. In our previous study, the use of RT-qPCR may have allowed the detection of low rRNA levels, leading to an increased detection rate of *E. faecalis* by that method. Moreover, the discrepancy between the present findings for streptococci when compared to our previous findings for *E. faecalis* may reflect differences in bacterial susceptibility to endodontic treatment procedures. The higher detection rate of *E. faecalis* by rRNA-based methods may be a consequence of higher amounts of viable *E. faecalis* in post-treatment samples due its resistance to endodontic procedures. Since the status of bacterial species after treatment may range from inactive/dead cells to cells with high metabolic activity, the use of rRNA-based molecular methods would be advantageous to elucidate the potential etiological agents of persistent endodontic infections. This method would be even more relevant in studies assessing the viability of uncultivated or difficult-to-culture bacterial species.

One limitation of this study was the use of molecular assays that did not allow a quantitative analysis of streptococci cells present in root canal samples. The viable bacterial load persisting after endodontic procedures may impact treatment outcomes.^19^ Therefore, the use of quantitative molecular methods would be imperative in endodontic microbiology studies. Because the absolute number of bacterial cells is related to the number of rRNA genes, new strategies have been investigated to overcome the limitation of rDNA-based assays in detecting dead bacterial cells. One of those strategies is the use of propidium monoazide (PMA) coupled with qPCR (PMA-qPCR).^12,20^ However, the detection limit of PMA-qPCR assays (10^3^ CFU/mL) may impair their use in molecular studies assessing the antimicrobial efficacy of endodontic procedures.^12,20^ In this sense, an important strategy to obtain information on both bacterial viability and number of bacterial cells in endodontic samples would be the association of rRNA- and rDNA-based qPCR assays, as previously demonstrated.^15^

## Conclusion

In summary, our study revealed differences in the detection frequency of streptococci using rRNA-based RT-PCR and rDNA-based PCR. Compared to rRNA-based RT-PCR, the rDNA-based PCR assay showed a higher detection rate of streptococci. This finding confirmed previous studies, suggesting that DNA detection may not always be associated with active endodontic infection. Therefore, rRNA-based methods may be more suitable to identify functional bacteria that effectively contribute to persistent endodontic infections.

## References

[1] Siqueira JF, Rôças IN (2009). Diversity of endodontic microbiota revisited. J Dent Res.

[2] Rôças IN, Siqueira JF (2012). Characterization of microbiota of root canal-treated teeth with posttreatment disease. J Clin Microbiol.

[3] Rôças IN, Siqueira JF (2010). Identification of bacteria enduring endodontic treatment procedures by a combined reverse transcriptase-polymerase chain reaction and reverse-capture checkerboard approach. J Endod.

[4] Neves MA, Rôças IN, Siqueira JF (2014). Clinical antibacterial effectiveness of the self-adjusting file system. Int Endod J.

[5] Rôças IN, Siqueira JF (2011). In vivo antimicrobial effects of endodontic treatment procedures as assessed by molecular microbiologic techniques. J Endod.

[6] Sakamoto M, Siqueira JF, Rôças IN, Benno Y (2007). Bacterial reduction and persistence after endodontic treatment procedures. Oral Microbiol Immunol.

[7] Rôças IN, Provenzano JC, Neves MA, Siqueira JF (2016). Disinfecting effects of rotary instrumentation with either 2.5% sodium hypochlorite or 2% chlorhexidine as the main irrigant: a randomized clinical study. J Endod.

[8] Siqueira JF, Rôças IN, Paiva SS, Guimarães-Pinto T, Magalhães KM, Lima KC (2007). Bacteriologic investigation of the effects of sodium hypochlorite and chlorhexidine during the endodontic treatment of teeth with apical periodontitis. Oral Surg Oral Med Oral Pathol Oral Radiol Endod.

[9] Siqueira JF, Magalhães KM, Rôças IN (2007). Bacterial reduction in infected root canals treated with 2.5% NaOCl as an irrigant and calcium hydroxide/camphorated paramonochlorophenol paste as an intracanal dressing. J Endod.

[10] Siqueira JF, Rôças IN (2005). Exploiting molecular methods to explore endodontic infections: Part 1 - current molecular technologies for microbiological diagnosis. J Endod.

[11] Brundin M, Figdor D, Sundqvist G, Sjögren U (2013). DNA binding to hydroxyapatite: a potential mechanism for preservation of microbial DNA. J Endod.

[12] Pinheiro ET, Neves VD, Reis CC, Longo PL, Mayer MP (2016). Evaluation of the propidium monoazide-quantitative polymerase chain reaction method for the detection of viable Enterococcus faecalis. J Endod.

[13] Matsuda K, Tsuji H, Asahara T, Kado Y, Nomoto K (2007). Sensitive quantitative detection of commensal bacteria by rRNA-targeted reverse transcription-PCR. Appl Environ Microbiol.

[14] Pitkänen T, Ryu H, Elk M, Hokajärvi AM, Siponen S, Vepsäläinen A (2013). Detection of fecal bacteria and source tracking identifiers in environmental waters using rRNA-based RT-qPCR and rDNA-based qPCR assays. Environ Sci Technol.

[15] Pinheiro ET, Candeiro GT, Teixeira SR, Shin RC, Prado LC, Gavini G (2015). RNA-based assay demonstrated Enterococcus faecalis metabolic activity after chemomechanical procedures. J Endod.

[16] Rôças IN, Hülsmann M, Siqueira JF (2008). Microorganisms in root canal-treated teeth from a German population. J Endod.

[17] Brundin M, Figdor D, Sundqvist G, Sjögren U (2015). Preservation of Fusobacterium nucleatum and Peptostreptococcus anaerobius DNA after loss of cell viability. Int Endod J.

[18] Brundin M, Figdor D, Roth C, Davies JK, Sundqvist G, Sjögren U (2010). Persistence of dead-cell bacterial DNA in ex vivo root canals and influence of nucleases on DNA decay in vitro. Oral Surg Oral Med Oral Pathol Oral Radiol Endod.

[19] Siqueira JF, Rôças IN (2008). Clinical implications and microbiology of bacterial persistence after treatment procedures. J Endod.

[20] Loozen G, Boon N, Pauwels M, Quirynen M, Teughels W (2011). Live/dead real-time polymerase chain reaction to assess new therapies against dental plaque-related pathologies. Mol Oral Microbiol.

